# Reconsidering the Role of Nurse Practitioners in Japan: What Direction Should Japanese Nurse Practitioners Aim for?

**DOI:** 10.7759/cureus.52936

**Published:** 2024-01-25

**Authors:** Ryoko Yamauchi, Ryuichi Ohta, Chiaki Sano

**Affiliations:** 1 Rehabilitation, Kanagawa Rehabilitation Hospital, Atsugi, JPN; 2 Community Care, Unnan City Hospital, Unnan, JPN; 3 Community Medicine Management, Shimane University Faculty of Medicine, Izumo, JPN

**Keywords:** healthcare policy, aging population, nursing education, task shifting in healthcare, healthcare delivery, nurse practitioner

## Abstract

This article explores the dynamic role of nurse practitioners in Japan, contextualized against an aging population and declining birth rates. It emphasizes the imperative for Japanese nurse practitioners to broaden their scope of practice to effectively meet the nation's diverse healthcare demands within a constrained resource framework. The study highlights the critical need for Japan to align its nurse practitioners’ training with international educational standards, advocating for a graduate-level curriculum that blends in-depth theoretical knowledge with practical skillsets. Central to the discourse is task shifting, wherein nurse practitioners progressively undertake duties traditionally associated with physicians. This expansion of roles requires meticulous evaluation to ensure its contribution to the efficacy of the healthcare system. The article identifies nurse practitioners as pivotal in team-based medical care, proficient in merging medical and nursing perspectives, and essential in facilitating communication and coordination among varied medical professionals. Comparative analysis of international nurse practitioner practice models reveals a necessity for Japan to enhance the scope and responsibilities of its nurse practitioners. Furthermore, the paper addresses the need for a comprehensive reevaluation of Japan's legal and policy framework concerning clinical nursing, aiming to redefine roles and responsibilities more distinctly. The article advocates for systemic reforms, particularly in education and multi-professional collaboration. These changes are deemed vital for Japanese nurse practitioners to respond to evolving healthcare needs effectively, ultimately elevating the standard of healthcare provision in Japan for a global audience.

## Editorial

In Japan, where the society is facing an aging population and declining birth rates, it is essential to meet the diverse healthcare and welfare needs of the population with limited resources [1]. This necessitates promoting effective and efficient team-based medical care, including task shifting and sharing among various professions [1]. In nursing, roles are expected to be expanded through specific actions and task shifting/sharing, and multiple qualification systems have emerged [2]. In 2008, in response to social issues, such as regional disparities in medical care provision, three-minute outpatient consultations, and the shuffling of emergency patients, Japan began training nurse practitioners as one measure to provide fair, effective, and timely medical care to those in need [2]. This was followed by the establishment in 2014 of a training system for nurses involved in specific actions, which started in 2015 [2].

The emergence of nurse practitioners and those who have completed specific action training is mainly due to expanding roles in response to changing medical needs, advances in medical care decreased medical caregivers due to a declining birth rate, and reforms in how doctors work [3]. Therefore, the primary role of nurse practitioners is currently seen as "task shifting," substituting for some of the doctor's work. This may not always be an effective role distribution. The training of nurse practitioners is unified at the master's level in graduate school, aligned with international standards [3]. While they acquire a certain level of medical knowledge and skills, the final qualification of nurse practitioners is still that of a nurse. Under current Japanese law, they cannot bear the same responsibilities as doctors [3].

Nurse practitioners have a foundation as nurses and should establish a new role or take on part of the doctor's work based on this foundation. They can view patients from medical and nursing perspectives, have a broad view of patient information, and thus have a high capacity for coordination [2]. As such, they are expected to act as "key persons in team medicine" and "gatekeepers of community medicine." In clinical settings, task shifting is often more visible and gets more attention [4]. Still, Japanese nurse practitioners should strengthen interprofessional communication and play a central role in team medicine. This direction requires a new approach to improve the quality of medical care, not just following the trend of task shifting [4].

In contemporary Japanese medical systems, the role of nurse practitioners is increasingly essential. However, this role varies by country, and especially in Japan, there are significant differences in qualification levels and job content compared to other countries [5]. The scope of practice for nurse practitioners varies by country and state, and in some states, they have the authority to diagnose, treat, and prescribe medications and open clinics. Nurse practitioners often fall under medical insurance and generally bill similarly to doctors, and their role in primary care can reduce healthcare costs [5]. Comparisons with foreign nurse practitioners may be limited due to different medical systems and cultures.

In Japan, which has universal health insurance, medical insurance does not cover nurse practitioners’ interventions [2]. Choosing nurse practitioners over doctors for daily treatment does not reduce medical costs, and nurse practitioners do not have the same authority as doctors, leading to ambiguous responsibilities [3]. In this context, the role of nurse practitioners in Japan needs to be reevaluated and redefined within the unique Japanese medical system [4]. Furthermore, reconsidering the competencies of nurse practitioners from the perspective of improving the quality of medical care and emphasizing their role in chronic care is essential [2]. Nurse practitioners, with their specialized support for life and their role as "key persons in team medicine" and "gatekeepers of community healthcare," have the potential to provide higher-quality medical care [3]. Unlike the unified university education for doctors, nursing education varies widely, impacting the specialization and quality improvement of the nursing profession. The cooperative master's level graduate school education for nurse practitioners contributes to higher specialization and quality improvement through deep academic knowledge.

In conclusion, the role of Japan's nurse practitioners needs to evolve and be revitalized according to contemporary medical needs. This requires reform in education, redefinition of job content, and strengthening interprofessional collaboration. Through these initiatives, nurse practitioners can play a more central and influential role in Japan's medical system.
